# Anti-inflammatory deficiencies in neutrophilic asthma: reduced galectin-3 and IL-1RA/IL-1β

**DOI:** 10.1186/s12931-014-0163-5

**Published:** 2015-01-24

**Authors:** Peng Gao, Peter G Gibson, Katherine J Baines, Ian A Yang, John W Upham, Paul N Reynolds, Sandra Hodge, Alan L James, Christine Jenkins, Matthew J Peters, Jie Zhang, Jodie L Simpson

**Affiliations:** Department of Respiratory Medicine, The Second Affiliated Hospital of Jilin University, Changchun, Jilin China; Department of Respiratory and Sleep Medicine, Hunter Medical Research Institute, New Lambton Heights, NSW Australia; Priority Research Centre for Asthma and Respiratory Disease, The University of Newcastle, Callaghan, NSW Australia; Woolcock Institute of Medical Research, Glebe, NSW Australia; School of Medicine, The University of Queensland, Brisbane, QLD Australia; Department of Thoracic Medicine, The Prince Charles Hospital, Brisbane, QLD Australia; Department of Respiratory Medicine, Princess Alexandra Hospital, Brisbane, QLD Australia; Department of Thoracic Medicine, Royal Adelaide Hospital, Adelaide, SA Australia; Lung Research Laboratory, Hanson Institute, Adelaide, SA Australia; Department of Pulmonary Physiology and Sleep Medicine, Sir Charles Gairdner Hospital, Perth, WA Australia; School of Medicine and Pharmacology, The University of Western Australia, Perth, WA Australia; Respiratory Trials, The George Institute for Global Health, Sydney, NSW Australia; Australian School of Advanced Medicine, Macquarie University, Sydney, NSW Australia; Department of Thoracic Medicine, Concord General Hospital, Concord, NSW Australia

**Keywords:** Asthma, Galectin-3, Induced sputum, Neutrophil, Macrophage, IL-1β, lectin

## Abstract

**Background:**

Galectin-3 (gal-3), a member of the β-galactoside-binding animal lectins, is involved in the recruitment, activation and removal of neutrophils. Neutrophilic asthma is characterized by a persistent elevation of airway neutrophils and impaired efferocytosis. We hypothesized that sputum gal-3 would be reduced in neutrophilic asthma and the expression of gal-3 would be associated with other markers of neutrophilic inflammation.

**Methods:**

Adults with asthma (n = 80) underwent a sputum induction following clinical assessment and blood collection. Sputum was dispersed for a differential cell count and ELISA assessment of gal-3, gal-3 binding protein (BP), interleukin (IL)-1β, IL-1 receptor antagonist (RA), IL-8 and IL-6. Gal-3 and gal-3BP immunoreactivity were assessed in mixed sputum cells.

**Results:**

Sputum gal-3 (median, (q1,q3)) was significantly reduced in neutrophilic asthma (183 ng/mL (91,287)) compared with eosinophilic (293 ng/mL (188,471), p = 0.021) and paucigranulocytic asthma (399 ng/mL (213,514), p = 0.004). The gal-3/gal-3BP ratio and IL-1RA/IL-1β ratio were significantly reduced, while gal-3BP and IL-1β were significantly elevated in neutrophilic asthma compared with eosinophilic and paucigranulocytic asthma.

**Conclusion:**

Patients with neutrophilic asthma have impairment in anti-inflammatory ratio of gal-3/gal-3BP and IL-1RA/IL-1β which provides a further framework for exploration into pathologic mechanisms of asthma phenotypes.

**Electronic supplementary material:**

The online version of this article (doi:10.1186/s12931-014-0163-5) contains supplementary material, which is available to authorized users.

## Background

Asthma is a heterogeneous chronic inflammatory airway disease characterized by airway hyperresponsiveness (AHR) and reversible airway obstruction [1]. While the allergen-induced Th2-lymphocyte, IL-5 mediated, eosinophilic response in asthma is now well characterized [1], recent research has shown that up to 50% of all asthma cases show no evidence of eosinophilic inflammation, termed non-eosinophilic asthma, and a subgroup of these have a persistence of airway neutrophilia, termed neutrophilic asthma [2,3]. The non-eosinophilic inflammatory phenotypes exhibit a poor response to inhaled corticosteroid [4,5]. We have previously described four distinct subtypes of asthma based on the inflammatory cells count in induced sputum, namely, neutrophilic asthma (NA), paucigranulocytic asthma (PGA), mixed eosinophilic and neutrophilic asthma (mixed granulocytic asthma (MGA)) and eosinophilic asthma (EA). In recent years, many studies have demonstrated that each subtype has a distinct mechanism and a different response to therapy [6-8]. In eosinophilic asthma, biomarkers such as eosinophilia (blood, sputum), FeNO and periostin can indicate corticosteroid responsiveness [9], but for non-eosinophilic asthma further work is needed to characterize the chronic inflammation and inflammatory cell accumulation to provide insights into effective, individual and targeted therapeutic options and also to identify potential biomarkers that can indicate phenotype and potential treatment response.

Galectins are a family of β-galactoside-binding animal lectins which function in a variety of biological processes including inflammation and allergic pathologies [10,11]. The extracellular and intracellular concentrations and surface expression of galectin-3 (gal-3) are increased during inflammation [12,13]. Gal-3 has an important role in the recruitment, activation and removal of neutrophils and can induce the release of IL-8, a key cytokine involved in neutrophil recruitment and activation [14]. Data from gal-3 null mice show reduced neutrophil recruitment during infection [13] and gal-3 increases uptake of apoptotic neutrophils. Therefore any alteration to gal-3 function may impact on the ability to remove apoptotic neutrophils from the site of inflammation [15]. This has several consequences as reduced removal of apoptotic cells can result in the release of damaging enzymes and oxidants which can promote persistence of inflammation. In COPD, there are reduced levels of bronchoalveolar lavage gal-3 which when restored, improved macrophage efferocytosis supporting an important role for gal-3 in the airways in chronic non-eosinophilic airway diseases [16].

Galectin-3 binding protein (gal-3BP), also known as the tumor-associated antigen 90 K, a native ligand of gal-3, is a member of the macrophage scavenger receptor cysteine-rich domain superfamily. Investigations have shown that gal-3BP is involved in normal tissue homeostasis including promoting cytokine secretion [17,18], modulating the inflammatory response [19] and participating in wound healing [20]. Local and systemic levels of gal-3BP are elevated in asthma and inhibit Th2 cytokine transcription while promoting the production of IL-6. In addition, systemic gal-3BP levels in asthma are significantly negatively associated with peripheral blood eosinophil counts [21]. Recent studies indicate that gal-3 and its binding protein might be involved in the pathophysiological mechanisms of asthma [12,21] and in particular non-eosinophilic asthma. Since gal-3BP binds active gal-3 and effectively reduces its function, a persistent airway neutrophilia could result from either reduced levels of gal-3, increased gal-3BP, or both.

We hypothesized that levels of gal-3 and the ratio of gal-3 to gal-3BP would be reduced in neutrophilic asthma. Similarly, we also hypothesized that the ratio of IL-1RA to inflammatory IL-1β would also be reduced in neutrophilic asthma and that sputum gal-3 levels would be associated with markers of neutrophilic inflammation.

## Methods

### Participant population

Eligible adults with asthma that was sub-optimally controlled were recruited from tertiary care centers around Australia. A group of 80 adults with asthma were assessed and sputum collected for the measurement of a panel of biomarkers of inflammatory phenotype including gal-3, gal-3BP, IL-1RA, IL-1β, IL-6 and IL-8. Serum was available in a sub-group of participants for the assessment of gal-3 and gal-3BP (n = 57).

The diagnosis of asthma was established using the American Thoracic Society guidelines based upon current episodic respiratory symptoms (past 12 months), clinical diagnosis and evidence of variable airflow obstruction [22]. Participants were all prescribed inhaled corticosteroids (ICS) or combination inhaled corticosteroid/long acting bronchodilator therapy (ICS/LABA)but remained not well controlled (asthma control questionnaire 6 (ACQ6) >0.7) despite receiving this therapy. All participants underwent a clinical assessment which included history of smoking, respiratory symptoms, skin prick allergy testing and sputum induction and gave written informed consent. Ethical approval was granted by Hunter New England Human Research Ethics Committee approval number 08/11/19/3.03.

Participants were excluded if they had a post-bronchodilator FEV_1_ < 40% predicted, were a current smoker or an ex-smoker who had ceased smoking within the last year. Those with significant smoking related airspace disease (ex-smokers with more than 10 pack year history and DLCO/VA <70% predicted OR a smoking history >10 pack years with an exhaled carbon monoxide >10 ppm) were also excluded. Participants were assessed during a stable phase of disease with no treatment with oral corticosteroids or antibiotics, no exacerbations and no change in asthma medications over the previous four weeks.

### Sputum and blood collection

Sputum induction and processing were performed as previously described [23]. Briefly, a fixed sputum induction time of 15 minutes with hypertonic saline (4.5%) was used for all participants. For inflammatory cell count, sputum cells were dispersed using dithiothreitol (DTT) and cells resuspended in phosphate-buffered saline (PBS) [23]. The suspension was filtered and a total cell count (TCC) of leucocytes and cell viability was performed. Cytospins were prepared, stained (May-Grunwald Giemsa) and a differential cell count obtained from 400 non-squamous cells. The quality of induced sputum samples was assessed and considered adequate for samples with fewer than 50% squamous epithelial cells and more than 40% cell viability.

Blood was collected in a 9 mL EDTA tube, mixed gently and then centrifuged at 700 g for 10 minutes at room temperature and serum was stored at -80°C.

### Biomarkers of neutrophilic inflammation

The levels of sputum IL-1β, IL-1RA, IL-6, IL-8, gal-3 (R&D Systems; Minneapolis, MS, USA) and gal-3BP (eBioscience; San Diego, CA, USA) were measured by ELISA according to the manufacturer’s instructions. We have established the validity of IL-RA, gal-3 and gal-3BP and IL-6 assays for the use in induced sputum and IL-8 and IL-1β validations have been reported elsewhere [24,25]. The addition of DTT to the commercial standard showed no effect on the ELISA and all mediators showed better than 80% recovery in spiking experiments. These data are available in Additional file 1.

### Asthma subtype classification

The granulocyte cut-off values used were 3% for sputum eosinophils and 61% for sputum neutrophils [26,27]. Individual patients were classified as eosinophilic asthma (EA) with sputum eosinophils ≥3% of total cells, as neutrophilic asthma (NA) with neutrophils ≥61%, as paucigranulocytic asthma (PGA) with eosinophils ≤3% and neutrophils <61% and as mixed granulocytic asthma (MGA) with eosinophils ≥3% and neutrophils ≥61%.

### Sputum immunocytochemistry

Sputum immunocytochemistry was performed as previously described (25). Briefly, cytospins were fixed in PLP fixative, dried and coated in 15% sucrose and stored at -20°C. Thawed cytospins were washed, permeabilized and blocked. Primary antibodies anti-galectin-3 (EP2775Y) and anti-LGALS3BP (3G8) (Abcam, Cambridge, UK) were added followed by secondary donkey Alexa Fluor® antibodies matched for species (Life Technologies, Carlsbad, CA, USA). Cells were mounted using ProLong® Gold Antifade Mountant with DAPI (Life Technologies). Slides were observed on an Axio Imager A1 epifluorescence microscope (Carl Zeiss MicroImaging Inc, Thornwood, NY, USA) under fluorescent optics and pictures taken using an Olympus DP70 digital microscope camera (Olympus America, Centre Valley, PA, USA). Pictures were observed visually with the inflammatory mediators represented by the colours green (gal-3) and red (gal-3BP). Colocalization was assessed as both gal-3 and gal-3BP being present in the same location identified by the presence of a yellow colour.

### Statistics

IBM SPSS Statistics 17.0 was used for statistical analysis. Normally distributed data were summarized as the mean and standard deviation (SD) and more than two groups compared using ANOVA with least significant difference (LSD) post hoc testing or Student’s t-test for two groups. All levels of inflammatory mediators were log-transformed and normal distribution was checked thereafter and the analysis was conducted by using analysis of variance with LSD post hoc test and adjusted for age and body mass index (BMI) because there were significant correlations between age, BMI and some inflammation mediators, such as gal-3BP and IL-1β. Non-parametric data were reported as the median and interquartile range (IQR) and analyzed by Kruskal-Wallis test followed by Bonferroni correction or the Mann-Whitney U test. Spearman’s rank correlation coefficient was used to test correlations and also adjusted for age and BMI. Categorical variables were analyzed by Chi-squared test. A p value of <0.05 was considered statistically significant.

Logistic regression was used to establish whether the ratio of gal-3 to gal-3BP was a predictor of asthmatic phenotypes. We examined seven variables on the basis of the strength of the univariate associations (p < 0.2): atopy, FEV_1_, FVC and ratio of gal-3 to gal-3BP. Age, sex and BMI were included and other variables were added stepwise in a multivariable logistic regression model.

## Results

### Gal-3 and gal-3BP in asthma inflammatory phenotypes

Participants’ sex, age, BMI, smoking history, atopy and ACQ6 were similar among the four asthma subgroups [Table 1]. Participants with PGA had the best lung function compared to other groups. Those with NA had the greatest sputum total cell count and number of neutrophils compared with the other groups, followed by MGA. There were higher numbers of eosinophils in MGA and EA than in NA and PGA, while EA had a decreased number of macrophages and lymphocytes compared with PGA and MGA respectively.

**Table 1:** **Clinical characteristics and sputum cell numbers by asthma inflammatory subtypes**

	**MGA**	**NA**	**PGA**	**EA**	**p value**
N	5	18	29	28	
Sex (m/f)	4/1	8/10	15/14	12/16	0.461
Age (y)	64 ± 11	62 ± 12	58 ± 14	61 ± 8	0.518
BMI	28 ± 3.6	30 ± 6.7	33 ± 7.0	30 ± 6.8	0.270
Atopy (y/n)	4/0	19/6	22/6	14/1	0.390
Ex-smoker (y/n)	3/2	11/17	10/19	6/11	0.741
Pack years	3 (2.3,17)	28 (14,49)	9.3(1,42)	15 (3.6,26)	0.772
FEV_1_ predicted (%)	64 (51,64)	65 (51,71)	80 (71,84) ^¶*§^	69 (54,78)	0.007
FEV_1_/FVC (%)	72 (67,79)	77 (68,86)	86 (76,95)	78 (71,88)	0.157
ACQ6	1.2 (1.2,1.3)	1.8 (1.3,2.3)	1.7 (1.3,2.2)	1.5 (1.2,2.4)	0.264
ICS/LABA, n (%)	5 (100%)	18 (100%)	27 (93%)	2 (86%)	0.291
ICS dose	1000 (1000,2000)	2000 (1000,2000)	1000 (800,2000)	1600 (800,2000)	0.767
Total cell counts (10^6^/mL)	8.2 (8.1,17)	12 (8.8,29)^#▽^	5.4 (3.8,11)	6.2 (2.5,9.5)	0.001
Viability (%)	90 (85,91)	89 (83,93)	79 (64,86)	74 (62,84)	0.001
Neutrophils (10^6^/mL)	5.8 (5.1,13)^#▽^	10 (6.7,24)^¶#▽^	1.6 (1.0,3.3)	1.9 (0.5,3.5)	<0.001
Eosinophils (10^6^/mL)	0.4 (0.3,1.5)^†#^	0.1 (0.0,0.2)	0.0 (0.0,0.1)	0.6 (0.3,2.0) ^#§^	<0.001
Macrophages (10^6^/mL)	2.3 (2.0,2.7)	2.4 (1.5,3.9)	3.2 (1.9,6.0)^▽^	1.6 (1.0,3.5)	0.014
Lymphocytes (10^6^/mL)	0.2 (0.1,0.2)^*^	0.1 (0.0,0.3)	0.0 (0.0,0.04)	0.0 (0.0,0.1)	0.009
Serum gal-3 (ng/mL)	2.1 (2.0-2.1) N = 2	1.4 (1.1-2.0) N = 13	1.5 (1.0-1.9) N = 21	1.4 (1.1-1.8) N = 21	0.480
Serum gal-3BP (mg/mL)	2.9 (2.8-3.1)	3.4 (3.3-5.8)	4.5 (2.9-6.0)	3.9 (2.9-5.2)	0.575

Data are expressed as mean ± SD or median (IQR). Data were analyzed by ANOVA or Kruskal-Wallis. MGA: mixed granulocytic asthma; NA: neutrophilic asthma; PGA: paucigranulocytic asthma; EA: eosinophilic asthma; BMI: body mass index; FEV_1_: forced expiratory volume in one second; FVC: forced vital capacity; ACQ6: asthma control questionnaire 6; ICS: inhaled corticosteroid; LABA: long-acting beta agonist; gal-3: galectin-3; gal-3BP: galectin-3 binding protein.

p < 0.05: ^¶^vs. MGA, † vs. NA, *vs. EA. p < 0.01: ^§^vs. NA, ^#^vs. PGA, ^▽^vs. EA.

Serum gal-3 and gal-3BP were similar across the inflammatory phenotypes [Table 1]. Due to the small numbers of participants with MGA further analyses are conducted among those with NA, PGA and EA.

### Inflammatory mediators by asthma inflammatory subtype

**Figure 1:**
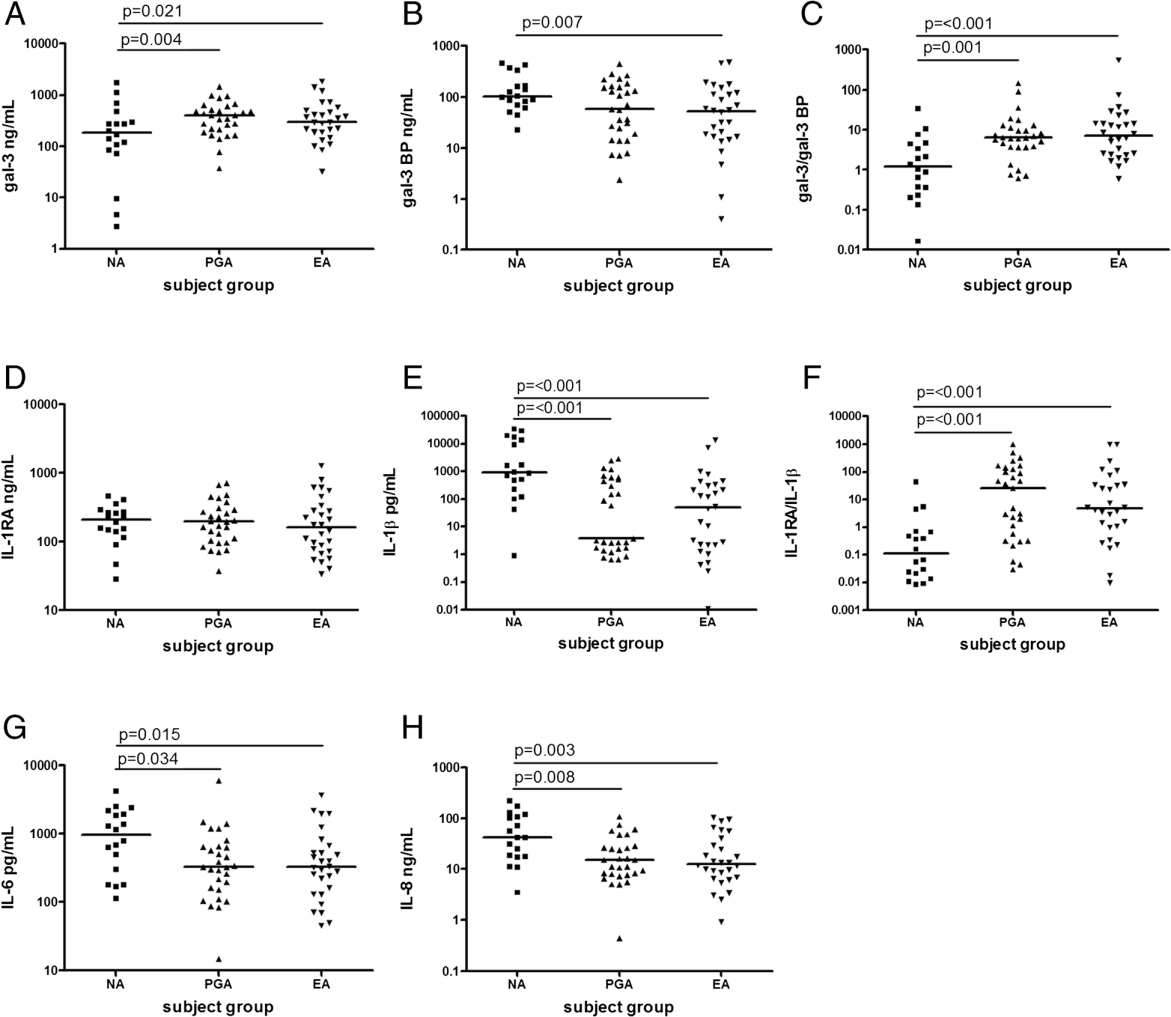
**Induced sputum concentrations of inflammatory mediators in asthmatic inflammatory subtypes.** Gal-3 **(A)** was decreased, gal-3BP **(B)** was increased and the gal-3 to gal-3BP ratio **(C)** was decreased in NA. IL-1RA **(D)** was not different, IL-1β **(E)** was increased and the IL-1RA to IL-1β ratio **(F)** was reduced in NA. IL-6 **(G)** and IL-8 **(H)** were both increased in NA. Group comparisons were conducted using analysis of variance with least significant difference (LSD) post hoc test after being log-transformed and adjusted by age and BMI. The horizontal bar denotes median value. NA; neutrophilic asthma, PGA; paucigranulocytic asthma, EA; eosinophilic asthma.

Sputum gal-3 was significantly reduced in NA compared with PGA and EA and gal-3BP was increased in NA compared with EA. The gal-3 to gal-3BP ratio was significantly reduced in participants with NA compared with EA and PGA. Participants with NA also had significantly increased concentrations of sputum IL-1β, IL-6 and IL-8 compared with PGA and EA. Although the level of IL-1RA was not different between the asthma inflammatory subtypes, the ratio of IL-1RA to IL-1β was significantly lower in NA compared with EA and PGA [Figure 1].

### Association between inflammatory mediators and clinical characteristics

Sputum gal-3 was negatively associated with the total number of sputum cells, the number of sputum neutrophils and lymphocytes while gal-3BP was positively associated with sputum total cells, neutrophils, macrophages and lymphocytes [Table 2].

**Table 2:** **Correlations between sputum inflammatory cells and sputum inflammatory mediators in asthma patients**

	**Total cells (×10** ^**6**^ **/mL)**	**Neutrophils (×10** ^**6**^ **/mL)**	**Macrophages (×10** ^**6**^ **/mL)**	**Lymphocytes (×10** ^**6**^ **/mL)**
Log gal-3	-0.333^#^	-0.412^*^	NS	-0.278^#^
Log gal-3BP	0.488^*^	0.378^#^	0.361^#^	0.254^#^
Log IL-1β	0.599^*^	0.607^*^	NS	0.425^*^
Log IL-8	0.520^*^	0.521^*^	NS	0.439^*^
Log IL-6	0.380^#^	0.338^#^	0.232^#^	0.255^#^

The data were analyzed by partial correlation, adjusted by age and BMI. *p < 0.001, ^#^p < 0.05, NS: not significant (p > 0.05). Gal-3: galectin-3; gal-3BP: galectin-3 binding protein; IL-1β: interleukin 1β; IL-8: interleukin 8; IL-6: interleukin 6.

Sputum IL-1β, IL-8 and IL-6 were all positively associated with the sputum total cell count, number of neutrophils and lymphocytes. While sputum IL-6 was significantly associated with the number of sputum macrophages. There were no significant relationships between IL-1RA and sputum inflammatory cell counts (data not shown). None of the sputum markers were associated with sputum eosinophil number (all p > 0.05).

Gal-3 had a significant inverse association with IL-1β and positive association with IL-6. Gal-3BP was significantly positively associated with IL-1β, IL-6, and IL-8. The typical pro-inflammatory mediators such as IL-1β, IL-6, and IL-8 were positively associated with each other. There were no significant correlations between IL-1RA and other mediators (data not shown).

Only IL-1RA had a significant relationship with FEV_1_ (r = 0.386, p < 0.001) and FVC (r = 0.332, p < 0.001) Table 3.

**Table 3:** **Correlations between sputum inflammatory mediators in asthma patients**

	**Log gal-3BP**	**Log IL-1β**	**Log IL-8**	**Log IL-6**
Log gal-3	NS	-0.238^#^	NS	0.251^#^
Log gal-3BP	N/A	0.570^*^	0.552^*^	0.532^*^
Log IL-1β	0.570^*^	N/A	0.701^*^	0.552^*^
Log IL-8	0.552^*^	0.701^*^	N/A	0.758^*^
Log IL-6	0.532^*^	0.552^*^	0.758^*^	N/A

Data were analyzed by the Partial Correlation test, adjusted by age and BMI. *p < 0.001, ^#^p < 0.05, NS: not significant (p > 0.05). Gal-3: galectin-3; gal-3BP: galectin-3 binding protein; IL-1β: interleukin 1β; IL-8: interleukin 8; IL-6: interleukin 6.

In participants with neutrophilic asthma sputum gal-3 levels were significantly positively associated with mean ACQ score (spearman’s r = 0.500, p = 0.049) but not with FEV_1_% predicted or FEV_1_/FVC (data not shown). There were no significant associations between sputum gal-3BP or the gal-3/gal-3BP with ACQ, FEV_1_ % predicted for FEV_1_/FVC (data not shown).

### Gal-3 and gal-3BP in sputum cells

**Figure 2:**
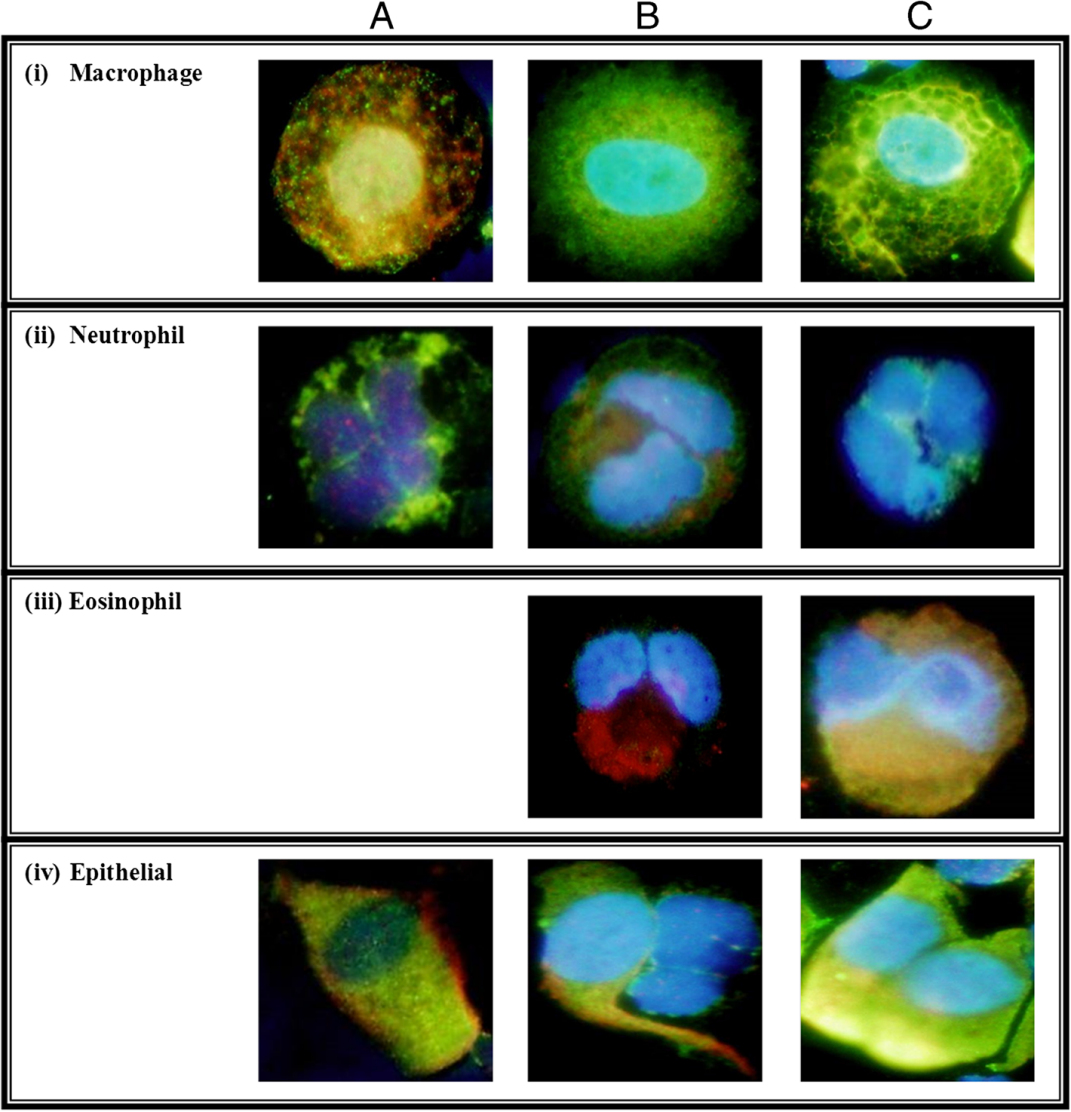
**Sputum cells from; A) neutrophilic, B) eosinophilic and C) paucigranulocytic asthma stained for gal-3 (green) and gal-3BP (red) with nuclear DAPI.**

Gal-3 and gal-3BP immunoreactivity was observed in sputum macrophages from all subtypes of asthma [Figure 2i]. Macrophages in neutrophilic asthma appeared to have less gal-3 staining compared with both eosinophilic and paucigranulocytic asthma and when gal-3 was present, it was colocalized with gal-3BP. Macrophages in EA and PGA appeared to have very intense staining for gal-3 and markedly less gal-3BP immunoreactivity. In the sputum neutrophils, there appeared to be less nuclear gal-3 in the participant with NA compared with EA and PGA [Figure 2ii].

### Logistic regression analysis to identify predictors of asthma phenotypes

The ratio of gal-3 to gal-3BP was an independent predictor of the presence of NA from EA and PGA after correcting for age, sex and BMI. The model reached statistical significance with an adjusted R^2^ of 0.322, p = 0.019. Full details of the regression can be found in Table S1 of Additional file 2.

### Effect of asthma medication

We compared the sputum inflammatory mediator levels in those taking ICS only with those taking combination ICS/LABA therapy. Sputum IL-β levels were very low in those participants taking ICS only and significantly reduced compared to ICS/LABA. There were no differences in any other mediators. Similarly, there was no difference in any mediator in those participants taking very high doses of ICS compared with those taking lower doses of ICS [Table 4].

**Table 4:** **Analysis of mediators according to ICS and/or LABA use and ICS dose categories**

	**ICS group**	**ICS/LABA group**	**ICS dose <1000**	**ICS dose <2000**	**ICS dose ≥2000**	**P value**	**P’ value**
N	6	74	24	19	37		
gal-3 (ng/mL)	356 (293,471)	274 (166,471)	318 (210,493)	289 (162,420)	273 (163,471)	0.454	0.526
gal-3BP (ng/mL)	43 (25,115)	71 (27,161)	58 (21,158)	75 (23,196)	71 (33,142)	0.559	0.930
gal-3/gal-3BP	10.3 (2.4,19.1)	5.2 (1.7,10.6)	6.2 (2.3,23)	5.6 (3.0,9.3)	4.7 (1.3,9.5)	0.352	0.501
IL-1RA (ng/mL)	80 (67,233)	176 (103,288)	200 (97,323)	161 (111,286)	162 (102,271)	0.100	0.855
IL-1β (pg/mL)	1 (0.6,3)	156 (3,694)	157 (2.8,734)	53 (1.5,732)	157 (3.5,603)	0.030	0.733
IL-1RA/IL-1β	73 (35,122)	2.1 (0.23,41)	3.3 (0.3,43)	5.34 (0.2,90)	1.1 (0.2,26)	0.144	0.755
IL-6 (pg/mL)	447 (397,502)	337 (169,1186)	501 (311,1140)	400 (197,903)	326 (178,1195)	0.770	0.874
IL-8 (ng/mL)	14.7 (11.0,40.6)	17.5 (8.5,50	18 (11.5,56)	25 (7.2,51)	14 (9.3,31)	0.985	0.819

Data are expressed as median (IQR). P value: ICS group vs. ICS/LABA group, data were analyzed by Mann-Whitney U test. P’ value: among different ICS dose categories, data were analyzed by Kruskal-Wallis. ICS: inhaled corticosteroid; LABA: long-acting beta agonist; gal-3: galectin-3; gal-3BP: galectin-3 binding protein; IL-1RA: IL-1 receptor antagonist; IL-1β: interleukin 1β; IL-6: interleukin 6; IL-8: interleukin 8.

We then conducted a multiple linear regression to investigate the factors that are independently associated with sputum gal-3 levels. The dose of ICS was not associated with sputum gal-3 levels (p = 0.289) and therefore was not included in the final model. The model was highly significant with BMI, sputum bronchial epithelial cell proportion, sputum IL-1β and previous smoking being independently associated with sputum gal-3 levels [Table 5].

**Table 5:** **Multivariate linear regression outcomes with dependent variable sputum supernatant gal-3 levels**

**Variable**	**Coefficient**	**SE**	**P**	**95% confidence interval**
Age	-0.002	0.004	0.686	-0.010 to 0.007
Sex	-0.022	0.094	0.813	-0.212 to 0.167
BMI	-0.201	0.007	0.007	-0.035 to -0.006
Bronchial epithelial cells, %	0.019	0.009	0.041	0.008 to 0.037
Ever smoked	0.210	0.102	0.042	0.008 to 0.413
Il-1β pg/mL	-3.8×10^-5^	8.0×10^-6^	<0.001	-5.8×10^-5^ to -2.2×10^-5^
Constant	3.114	0.385	<0.001	2.347 to 3.882

BMI: Body Mass Index; IL-1β: interleukin 1β.

## Discussion

In this study we found that both gal-3 and gal-3BP protein levels were more abundant in sputum than in serum. This may suggest a functionally important difference or it may reflect alterations in proteins during sputum processing. The lowest concentrations of sputum gal-3 were seen in neutrophilic asthma compared with paucigranulocytic and eosinophilic asthma and the highest concentrations of gal-3BP were seen in neutrophilic asthma. Binding of gal-3 to its binding protein would be expected to reduce the functional activity of gal-3 and this overall effect can be summarized as the gal-3/gal-3BP ratio which was also significantly lower in neutrophilic asthma. Together, these results suggest that the balance of gal-3 and gal-3BP is a determinant of airway neutrophilia, where gal-3BP appears to be a positive regulator and gal-3 may be a negative regulator of airway neutrophilia. These conclusions are supported by the direction of correlations between gal-3 and gal-3BP and sputum total cells, the number of neutrophils and lymphocytes, the correlation with other pro-inflammatory mediators and the results of the logistic regression analysis which identified the gal-3/gal-3BP ratio as a significant independent predictor of the neutrophilic asthma phenotype.

Reduced gal-3 levels in neutrophilic asthma may alter neutrophil function and accumulation. Addition of gal-3 to leukocytes increases uptake of apoptotic neutrophils and therefore any alteration to gal-3 expression may impact on the ability to remove apoptotic neutrophils from the site of inflammation [15]. This has several consequences, as poor removal of apoptotic cells can result in release of damaging enzymes and oxidants which can promote persistence of inflammation. This means that when there is relatively less gal-3 in the airway in neutrophilic asthma, neutrophil persistence could occur by reduced clearance. We have recently shown that efferocytosis is reduced in patients with non-eosinophilic asthma [28] of which neutrophilic asthma constitutes around 40% of the population. Similarly, in COPD where airway gal-3 is also reduced, addition of exogenous gal-3 improved the ability of macrophages to efferocytose apoptotic epithelial cells [16], suggesting a restoration in the ability of airway macrophages to remove dead cells and therefore avoid cell necrosis. Our results are supported by a small study of patients with asthma and healthy controls where significantly reduced gal-3 mRNA expression was shown compared to healthy controls along with expression of gal-3 in sputum macrophages and neutrophils [29].

The binding of gal-3 to its soluble binding protein may explain the reduced levels of gal-3 observed in our study. Alternative explanations for the reduced gal-3 levels observed in neutrophilic asthma may be due to proteolytic cleavage of gal-3 by endogenous proteases and bacterial collagenases [13,30,31] which may include neutrophil elastase which is also elevated in neutrophilic asthma (24). These mechanisms require further exploration in future studies.

Gal-3 is involved in innate immune responses and recognition of bacteria. Higher gal-3 levels are associated with alternative macrophage activation [16,32] and classical activation of macrophages via LPS is associated with inhibition of gal-3 [33]. Interestingly, the classically activated macrophage phenotype is associated with corticosteroid resistant asthma suggesting that low gal-3 levels may identify those who are corticosteroid resistant [34]. When macrophages are deficient in gal-3 there is increased responses to LPS [35], reduced bacterial replication and increased expression of IL-1β, TLR2 and IL-6 [36], which are similar observations to those we have previously reported in patients with the neutrophilic subtype of asthma and COPD [25,37,38]. In the present study we observed a negative association between gal-3 and IL-1β levels in sputum supernatant suggesting that gal-3 may indeed be anti-inflammatory while IL-1β is known to be an important inflammatory mediator increased in the airways of patients with neutrophilic asthma [25].

Gal-3 and gal-3BP are expressed in many airway cell types including macrophages, eosinophils, neutrophils and mast cells [13,39,40]. We observed gal-3 and gal-3BP expression in airway macrophages and neutrophils however the pattern of expression was quite different in neutrophilic asthma compared with eosinophilic and paucigranulocytic asthma, supporting the protein data which suggest altered levels of gal-3 and gal-3BP in neutrophilic asthma. Expression of gal-3 was observed in both the nucleus and cytoplasm which is consistent with the variable subcellular location [41] and may indicate different processes in different asthma inflammatory phenotypes as have been observed for gal-3 in cancer [42]. Further studies may help identify the precise location of gal-3 in airway cells from patients with asthma and the role of galectin in specific inflammatory phenotypes.

Sputum levels of IL-1β were also significantly elevated in neutrophilic asthma, in agreement with our previous data [25,38] and we extend this by showing that levels of IL-1RA, the receptor antagonist for IL-1β, was not different between the asthma phenotypes. This suggests that the increased IL-1β observed in neutrophilic asthma is not due to reduced IL-1 receptor antagonism by IL-1RA. Similar IL-1RA levels in asthma inflammatory phenotypes also suggest an impairment of anti-inflammatory responses where increased IL-1RA could oppose the high IL-1β levels. When we compared the IL-1RA/IL-1β ratio it was significantly reduced in neutrophilic asthma suggesting an imbalance of available anti-inflammatory mediators similar to that observed with the gal-3/gal-3BP ratio. The sputum IL-1RA levels were significantly correlated positively with lung function in patients with asthma and negatively with IL-6, which supports IL-1RA being anti-inflammatory in the airways [43]. This is further supported by the observation that treatment of antigen-sensitized animals with IL-1RA inhibits *in vivo* airway hyperresponsiveness and airway inflammation [44].

Due to the cross-sectional design of this study we are not able to determine cause or effect. The strengths of the present study are the carefully characterized asthma population and the assessment of the inflammatory mediators in sputum. Further studies are needed to determine if gal-3 can improve the impaired phagocytosis identified in adults with non-eosinophilic asthma and to assess the response of gal-3 levels to inhaled corticosteroids.

## Conclusions

In conclusion, this study demonstrates for the first time, that the sputum gal-3/gal-3BP and IL-1RA/IL-1β ratios are associated with neutrophilic asthma and may suggest impairment of anti-inflammatory mediator expression. The results support the molecular heterogeneity of asthma and provide a further framework for exploration into pathologic mechanisms of asthma phenotypes, important for the development of more effective treatment.

## Additional files

Additional file 1:
**Mediator validation for assessment in induced sputum.** The addition of DTT to the commercial standard shows no effect on the ELISA and all mediators show better than 80% recovery in spiking experiments. Additional file 2:
**Logistic regression analysis to identify a predictor of asthma phenotypes.** Table S1 shows the logistic regression analysis to identify a predictor of asthma phenotypes.

## Abbreviations

Gal-3: Galectin-3
gal-3BP: galectin-3 binding protein
IL: Interleukin
ELISA: Enzyme-linked immunosorbant assay
BMI: Body mass index
AHR: Airway hyperresponsiveness
ACQ6: Asthma control questionnaire 6
FeNO: Fractional exhaled nitric oxide
FEV_1_: Forced expiratory volume in 1 second
FVC: Forced vital capacity
DLCO/VA: Diffusing capacity for carbon monoxide corrected for alveolar volume
ppm: parts per million
DDT: Dithiothreitol
PBS: Phosphate-buffered saline
TCC: Total cell count
SD: Standard deviation
ANOVA: Analysis of variance
LSD: Least significant difference
IQR: Interquartile range
OR: Odd ratio
CI: Confidence interval
ICS: Inhaled corticosteroid
COPD: Chronic obstructive pulmonary disease
NA: Neutrophilic asthma
EA: Eosinophilic asthma
PGA: Paucigranulocytic asthma
MGA: Mixed granulocytic asthma
