# Supplementary material for: Anti-inflammatory deficiencies in neutrophilic asthma: reduced galectin-3 and IL-1RA/IL-1β
Source: Respir Res. 2015 Jan 24;16(1):5. doi: 10.1186/s12931-014-0163-5 (PMC4314745; doi:10.1186/s12931-014-0163-5)
Supplement: Additional file 1: — Mediator validation for assessment in induced sputum. The addition of DTT to the commercial standard shows no effect on the ELISA and all mediators show better than 80% recovery in spiking experiments. [file 12931_2014_163_MOESM1_ESM.docx]

**Additional file 1**

**Methods**

***Mediator validation for assessment in induced sputum***

The recovery of recombinant mediator added to sputum samples prior to dispersion with dithiothreitol (DTT) was 151% for gal‑3, 84% for gal‑3BP, 161% for IL‑6 and 104% for IL‑1RA.

There was no effect of DTT on the commercial kit standard curves as shown in Figure 1.

**Figure S1 Mediator standard curves prepared using an equivalent DTT/PBS diluent to that in sputum supernatant samples**

A) Gal-3, B) Gal-3BP, C) IL-6, D) IL-1RA.
