# Supplementary material for: Anti-inflammatory deficiencies in neutrophilic asthma: reduced galectin-3 and IL-1RA/IL-1β
Source: Respir Res. 2015 Jan 24;16(1):5. doi: 10.1186/s12931-014-0163-5 (PMC4314745; doi:10.1186/s12931-014-0163-5)
Supplement: Additional file 2: — Logistic regression analysis to identify a predictor of asthma phenotypes. Table S1 shows the logistic regression analysis to identify a predictor of asthma phenotypes. [file 12931_2014_163_MOESM2_ESM.docx]

**Additional file 2**

**Results**

**Table S1 Logistic regression analysis to identify a predictor of asthma phenotypes**

|  | | Mixed Granulocytic Asthma | | | Paucigranulocytic Asthma | | | Eosinophilic Asthma | | | R^2^ | p value |
| --- | --- | --- | --- | --- | --- | --- | --- | --- | --- | --- | --- | --- |
|  |  | OR | CI (95%) | p value | OR | CI (95%) | p value | OR | CI (95%) | p value |  |  |
| Univariate | Age | 1.03 | 0.93-1.13 | 0.609 | 0.97 | 0.92-1.02 | 0.267 | 0.99 | 0.94-1.05 | 0.782 | 0.030 | 0.494 |
|  | BMI | 0.93 | 0.78-1.11 | 0.403 | 1.05 | 0.96-1.15 | 0.298 | 0.98 | 0.89-1.08 | 0.685 | 0.050 | 0.248 |
|  | Sex | 0.20 | 0.02-2.16 | 0.185 | 0.75 | 0.23-2.43 | 0.628 | 1.07 | 0.32-3.52 | 0.916 | 0.033 | 0.439 |
|  | Atopy | 0.50 | 0.04-7.0 | 0.607 | 0.479 | 0.09-2.7 | 0.403 | 0.42 | 0.07-2.4 | 0.321 | 0.015 | 0.765 |
|  | **FEV_1_** | **1.26** | **0.24-6.5** | **0.786** | **4.038** | **1.48-11.0** | **0.006** | **2.11** | **0.78-5.68** | **0.139** | **0.129** | **0.011** |
|  | FVC | 1.18 | 0.39-3.6 | 0.767 | 1.93 | 0.99-3.8 | 0.055 | 1.44 | 0.73-2.8 | 0.290 | 0.053 | 0.222 |
|  | **ACQ6** | **0.11** | **0.01-1.55** | **0.102** | **1.01** | **0.50-2.04** | **0.975** | **1.26** | **0.65-2.46** | **0.494** | **0.072** | **0.114** |
|  | Smoke | 0.96 | 0.88-1.04 | 0.313 | 0.99 | 0.95-1.03 | 0.606 | 0.98 | 0.93-1.02 | 0.259 | 0.064 | 0.551 |
|  | Gal-3 | 1.0 | 1.0-1.0 | 0.756 | 1.0 | 1.0-1.0 | 0.362 | 1.0 | 1.0-1.0 | 0.381 | 0.013 | 0.781 |
|  | **Gal-3BP** | **1.00** | **1.0-1.0** | **0.222** | **1.0** | **1.0-1.0** | **0.217** | **1.0** | **1.0-1.0** | **0.127** | **0.088** | **0.060** |
|  | **Gal-3/Gal-3BP ratio** | **1.09** | **0.95-1.3** | **0.237** | **1.11** | **1.0-1.25** | **0.061** | **1.12** | **1.0-1.25** | **0.052** | **0.107** | **0.028** |
| Multinomial logistic regression | Age | 1.042 | 0.93-1.17 | 0.476 | 1.046 | 0.98-1.12 | 0.209 | 1.043 | 0.97-1.12 | 0.253 | 0.322 | 0.019 |
|  | BMI | 0.894 | 0.74-1.09 | 0.263 | 1.026 | 0.93-1.13 | 0.608 | 0.943 | 0.85-1.05 | 0.261 |  |  |
|  | Sex | 0.214 | 0.02-2.93 | 0.248 | 2.918 | 0.62-13.8 | 0.177 | 1.867 | 0.42-8.26 | 0.411 |  |  |
|  | FEV_1_ | 1.560 | 0.22-11.0 | 0.655 | 8.693 | 2.15-35.1 | **0.002** | 3.670 | 0.94-14.3 | 0.062 |  |  |
|  | Gal-3/Gal-3BP ratio | 1.118 | 0.97-1.29 | 0.125 | 1.145 | 1.01-1.30 | **0.032** | 1.148 | 1.02-1.30 | **0.028** |  |  |

The reference category is neutrophilic asthma. OR: odd ratio; CI: confidence interval; BMI: body mass index; FEV_1_: forced expiratory volume in one second; FVC: forced vital capacity; ACQ6: asthma control questionnaire 6; Gal-3: galectin-3; Gal-3BP: galectin-3 binding protein.

**Table S2 Analysis of sputum inflammatory mediators according to ICS and/or LABA use and ICS dose categories**

|  | ICS group | ICS/LABA Group | ICS Dose  <1000 | ICS Dose  <2000 | ICS Dose  ≥2000 | p value | p’ value |
| --- | --- | --- | --- | --- | --- | --- | --- |
| N | 6 | 74 | 24 | 19 | 37 |  |  |
| Gal-3 (ng/mL) | 356 (293,471) | 274 (166,471) | 318(210,493) | 289(162,420) | 273(163,471) | 0.454 | 0.526 |
| Gal-3BP (ng/mL) | 43 (25,115) | 71 (27,161) | 58(21,158) | 75(23,196) | 71(33,142) | 0.559 | 0.930 |
| Gal-3/Gal-3BP ratio | 10.3 (2.4,19.1) | 5.2 (1.7,10.6) | 6.2(2.3,23) | 5.6(3.0,9.3) | 4.7(1.3,9.5) | 0.352 | 0.501 |
| IL-1RA (ng/mL) | 80 (67,233) | 176 (103,288) | 200(97,323) | 161(111,286) | 162(102,271) | 0.100 | 0.855 |
| IL-1 β(pg/mL) | 1 (0.6,3) | 156 (3,694) | 157(2.8,734) | 53(1.5,732) | 157(3.5,603) | 0.030 | 0.733 |
| IL-1RA/IL-1β ratio | 73(35,122) | 2.1(0.23,41) | 3.3(0.3,43) | 5.34(0.2,90) | 1.1(0.2,26) | 0.144 | 0.755 |
| IL-6 (pg/mL) | 447 (397,502) | 337 (169,1186) | 501(311,1140) | 400(197,903) | 326(178,1195) | 0.770 | 0.874 |
| IL-8 (ng/mL) | 14.7 (11.0,40.6) | 17.5 (8.5,50 | 18(11.5,56) | 25(7.2,51) | 14(9.3,31) | 0.985 | 0.819 |

Data are expressed as median (IQR). p value: ICS group vs. ICS/LABA group, data were analyzed by Mann-Whitney U test. p’ value: among different ICS dose, data were analyzed by Kruskal-Wallis.

ICS: inhaled corticosteroid; LABA: long-acting beta agonist; gal-3: galectin-3; gal-3BP: galectin-3 binding protein; IL-1RA: IL‑1 receptor antagonist; IL‑1β: interleukin 1β; IL‑6: interleukin 6; IL‑8: interleukin 8.
